# Supplementary material for: Bayesian refinement of protein structures and ensembles against SAXS data using molecular dynamics
Source: PLoS Comput Biol. 2017 Oct 18;13(10):e1005800. doi: 10.1371/journal.pcbi.1005800 (PMC5662244; doi:10.1371/journal.pcbi.1005800)
Supplement: S1 Archive — (BZ2) [file pcbi.1005800.s011.tar.bz2 › gromacs_4.62_bayesian_saxs_refi/share/html/online/g_densorder.html]

g\_densorder


| g\_densorder Main Table of Contents |

**VERSION 4.6  
Sat 19 Jan 2013**

---

### Description

A small program to reduce a two-phase density distribution
along an axis, computed over a MD trajectory
to 2D surfaces fluctuating in time, by a fit to
a functional profile for interfacial densities
A time-averaged spatial representation of the
interfaces can be output with the option -tavg

### Files

| option | filename | type | description |
| --- | --- | --- | --- |
| **-s** | topol.tpr | Input | Run input file: tpr tpb tpa |
| **-f** | traj.xtc | Input | Trajectory: xtc trr trj gro g96 pdb cpt |
| **-n** | index.ndx | Input | Index file |
| **-o** | Density4D.dat | Output, Opt. | Generic data file |
| **-or** | hello.out | Output, Opt., Mult. | Generic output file |
| **-og** | interface.xpm | Output, Opt., Mult. | X PixMap compatible matrix file |
| **-Spect** | intfspect.out | Output, Opt., Mult. | Generic output file |

### Other options

| option | type | default | description |
| --- | --- | --- | --- |
| **-[no]h** | bool | no | Print help info and quit || **-[no]version** | bool | no | Print version info and quit || **-nice** | int | 0 | Set the nicelevel || **-b** | time | 0 | First frame (ps) to read from trajectory || **-e** | time | 0 | Last frame (ps) to read from trajectory || **-dt** | time | 0 | Only use frame when t MOD dt = first time (ps) || **-[no]w** | bool | no | View output .xvg, .xpm, .eps and .pdb files || **-[no]1d** | bool | no | Pseudo-1d interface geometry || **-bw** | real | 0.2 | Binwidth of density distribution tangential to interface || **-bwn** | real | 0.05 | Binwidth of density distribution normal to interface || **-order** | int | 0 | Order of Gaussian filter, order 0 equates to NO filtering || **-axis** | string | Z | Axis Direction - X, Y or Z || **-method** | enum | bisect | Interface location method: bisect or functional || **-d1** | real | 0 | Bulk density phase 1 (at small z) || **-d2** | real | 1000 | Bulk density phase 2 (at large z) || **-tblock** | int | 100 | Number of frames in one time-block average || **-nlevel** | int | 100 | Number of Height levels in 2D - XPixMaps |

---

http://www.gromacs.org  
gromacs@gromacs.org
